# Comparison of the absolute and relative efficiencies of electroporation-based transfection protocols for *Plasmodium falciparum*

**DOI:** 10.1186/1475-2875-11-210

**Published:** 2012-06-21

**Authors:** Sandra Hasenkamp, Karen T Russell, Paul Horrocks

**Affiliations:** 1 Institute for Science and Technology in Medicine, Keele University, Staffordshire, ST5 5BG, UK

**Keywords:** Luciferase, Electroporation, Transfection efficiency, Malaria

## Abstract

**Background:**

Several electroporation protocols exist to transfect exogenous DNA into *Plasmodium falciparum.* To date, however, only a subjective analysis of their relative efficiencies has been reported.

**Methods:**

A time-course of luciferase reporter expression is used to provide an objective quantitative analysis of the absolute efficiency of three electroporation techniques; direct electroporation of ring stage infected erythrocytes, preloading of erythrocytes and a novel “double-tap” protocol that combines both approaches.

**Results:**

Preloading of erythrocytes shows a mean efficiency of 9.59x10^-6^, some 5–180 fold more efficient than matched experiments utilizing the “double-tap” and direct electroporation of ring stage infected erythrocytes alone, respectively.

**Conclusion:**

Evidence presented here provides the first quantitative assessment of both the absolute and relative efficiencies of a key molecular tool used to study the biology and pathogenesis of this important human pathogen.

## Background

Genetic modification of *Plasmodium falciparum* has provided an invaluable molecular tool to dissect the biology and pathogenesis of this important human pathogen [1-3]. Introduction of exogenous DNA, however, remains a relatively inefficient and costly enterprise, both in terms of reagents and time, and represents a major limiting step in any investigation. A range of approaches have been used to introduce exogenous DNA into *P. falciparum* infected erythrocytes (IE), including; chemical agents, biolistic delivery and electroporation, with electroporation proving the only consistently reliable approach [2,4]. Electroporation of ring stage IE was first described in 1995 by Wu *et al.,* who utilized a high voltage/low capacitance electric pulse [5]. Subsequently, a low voltage/high capacitance electric pulse was shown to be more efficient and remains in wide use throughout the community [6]. A final evolution, pioneered by Deitsch *et al.* was the pre-loading of erythrocytes with exogenous DNA, which were then mixed with late stage IE, where passive spontaneous uptake of DNA by parasites presumably occurs following invasion into the pre-loaded erythrocytes during the subsequent cycle of asexual intra-erythrocytic development [7].

To date, only a comparison of the relative efficiency of these different transfection approaches has been carried out; although these reveal a clear preference for the use of pre-loaded erythrocytes [4]. Of note, however, from these studies is that direct electroporation of IE is cited as having a much lower overall success rate of only 25% [4]. This contrasts with the experience of a number of laboratories, where overall success rates of 70-80% are routinely achieved. Moreover, determining the relative efficiency of these approaches relies on single, or at best two, subjective endpoints; typically either days post-transfection when recovering parasites are first observed or when the cultures reach a 1% parasitaemia [4,8].

Here a quantitative investigation of the absolute and relative efficiency of direct electroporation of IE and pre-loaded erythrocytes is reported. Using a luciferase reporter construct as the source of exogenous DNA, and an improved single-step lysis protocol for the qualitative measurement of luciferase levels from small samples of IE [9], a time-course for recovery of the transfected parasites post-electroporation can be carried out. In addition, a combination of these two electroporation approaches is investigated here. Ring stage IE are directly electroporated, allowed to mature for 24 hrs and then mixed with pre-loaded erythrocytes; termed here the “double-tap” technique. Using two concentrations of DNA in a “double-tap” also allows the transfection efficiency per unit concentration of DNA to be monitored.

## Methods

### Parasite culture

The *P. falciparum* clone AHEI was cultured using standard protocols in the presence of 5nM WR99210 [10]. Synchronization to prepare ring stage IE was carried out using 5% D-sorbitol [11]. Enrichment of late trophozoite/schizont IE was carried out using Plasmagel (Bellon, France) density gradient flotation [12]. Giemsa-stained thin blood smears were used to monitor parasite staging and parasitaemia. Immediately prior to transfection, AHEI were passaged into freshly isolated O^+^ erythrocytes (National Blood and Transfusion Service, UK), with this same single source of erythrocytes used in all experiments for the remainder of the study.

### Transfection and time-course of sampling

**Figure 1:**
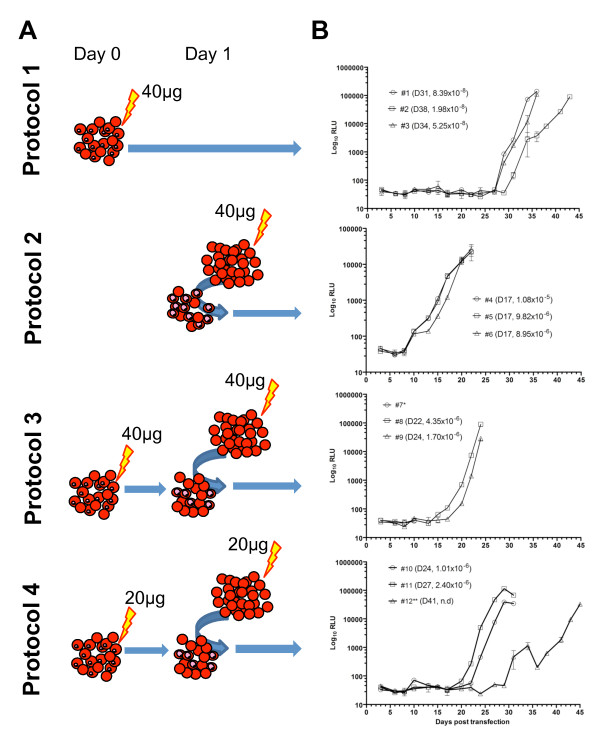
**Quantitative analysis of the relative efficiency of electroporation-based transfection techniques (A) Schematic representing the four protocols employed in this study.** Protocol 1, direct electroporation of 40 μg of plasmids pΔ1 and pINT into ring stage IE on Day 0; Protocol 2, preloading of 40 μg of plasmids into erythrocytes which are mixed with mature stage IE on Day 1; Protocols 3 (40 μg of plasmids) and 4 (20 μg of plasmids) use a combination of both Protocols 1 and 2 and is termed here the “double-tap” technique. (**B**) The timecourse of log_10_ RLU (Mean ± StDev, n = 3) over time for three independent transfections using each protocol. The key indicates the transfection tracking number (#), the days (D) post-transfection that an at least 1% asexual stage parasitaemia was directly observed microscopically and the mean transformation efficiency (parasites estimated to survive transfection N_o_/input parasites). *Transfection #7 was lost on day 3 with evidence of bacterial contamination of culture. **In transfection #12, the lid of the culture flask split between days 34–36, resulting in loss of low oxygen environment. No determination (n.d) of transfection efficiency was made for this experiment for this reason.

Large-scale preparation of plasmids pΔ1 and pINT was carried out using a commercial maxipreparation kit (Qiagen, UK). Plasmid preparations were pooled and the same common source of plasmid used in all subsequent experiments. pΔ1 contains a luciferase reporter gene flanked by 1418 bp and 647 bp of 5′ and 3′ *Pfpcna* sequences, respectively, in a pDC*att*P backbone to facilitate integration into the genome of the *P. falciparum* clone AHEI when cotransfected with pINT [10,13]. Four different protocols were investigated here, each performed in triplicate. Transfections 1–3 represent the direct electroporation of 40 μg each of pΔ1/pINT into ring stage IE (Figure 1A, Protocol 1) with transfections 4–6 using 40 μg each of pΔ1/pINT preloaded into erythrocytes (Protocol 2). Transfections 7–12 utilised a “double-tap” combination of these protocols; transfections 7–9 using double the amount (40 μg each of pΔ1/pINT twice, Protocol 3) of DNA compared to transfections 10–12 (20 μg each of pΔ1/pINT twice, Protocol 4). Thus, transfections 10–12 use the same overall amount of DNA as do transfections 1–6. As transfections 4–6 start 1 day later than the remainder, which all employ direct electroportation of ring IE on day 0, all calculations assume a start from day 1, although data is plotted from day 0 for common illustrative purposes.

Transfections of ring stage IE (transfections 1–3, 7–12) were carried out on 1.25x10^8^ parasites in a total volume of 400 μl in 1x cytomix [6]. Chilled 4 mm electroporation cuvettes were used with a GenePulser II electroporator (Biorad) set at 0.31KV and 950 μF. Immediately following electroporation, the IE were transferred to 10 ml of prewarmed complete medium, gassed (1% O_2_, 3% CO_2_, balance N_2_) and incubated at 37 °C. To preload erythrocytes, the appropriate amount of plasmid was added to 150 μl of packed erythrocytes in a total volume of 400 μl of 1x cytomix [7]. The same electroporation conditions as above were used. These preloaded erythrocytes were either mixed with the parasites transfected on day 0 (transfections 7–12) or with 2x10^8^ trophozoite/schizont stage IE (transfections 4–6). All transfection experiments were then matched to a 3% haematocrit (HCT), which was maintained over the remainder of the experiment.

From Day 2, drug selection was applied to the transfected culture by supplementing complete medium with 5nM WR99210, 2.5 μg/ml Blasticidin S and 125 μg/ml Neomycin; the latter two drugs representing selection for the pΔ1 and pINT plasmids, respectively. Throughout the selection procedure a common source of complete culture medium was used for all experiments. From Day 3, 200 μl of each transfected culture was periodically harvested approximately every 2 days (Mon, Wed, Fri). At this time, complete culture medium was replaced, washed erythrocytes added to maintain a 3% haematocrit and all flasks gassed before placing back at 37 °C. The 200 μl sample provided 3x40μl samples for determination of luciferase activity using an improved single-step lysis protocol [9] and IE to prepare a thin-blood smear for microscopic examination. To each 40 μl sample, 10 μl of 5x Passive Lysis Buffer (Promega, UK) was mixed, aliquoted into a well containing 50 μl of luciferase assay buffer (Promega, UK) on a 96-well white multiplate (Greiner, UK) and bioluminescence (in relative light units, RLU) measured for 2 sec in a GloMax Multi Detection System (Promega, UK).

### Analysis of growth and transfection efficiency

To correlate RLU with parasite number a standard curve was generated. Pfluc is an AHEI derivative in which the pΔ1 plasmid is integrated into the cg6 locus on chromosome 7 [9,13]. A serial three-fold dilution of a 1% starting parasitaemia at a 3% HCT was performed, maintaining a 3% HCT in each sample due to the quenching effect of released haemoglobin on luciferase bioluminescence [9]. Five replicates were prepared and the parasite number per sample plotted against mean log_10_ transformed RLU. Using a 1/250^th^ sampling volume (40 μl from 10 ml), an extrapolation to provide an estimate of the increase in parasite number over time in each transfected culture can be made. A logarithmic non-linear regression of parasite numbers over time (GraphPad Prism v5.01) allows the rate constant (k), i.e. the fold increase in parasite numbers per asexual cycle, to be determined. These values ranged from between 2.25-2.75 across the 10 experiments for which these could be plotted. Since there was no correlation between the rate constant and the protocol employed, a median of 2.5-fold increase in parasite numbers per cycle was used in all subsequent calculations. Using a generalised formula for logarithmic growth (N = N_o_e^kt^, where N represents the numbers of parasite following t cycles of growth at the rate constant k from an initial population of N_o_) the number of parasites that were successfully initially transfected could be estimated and are indicated here as a proportion of the input parasite numbers to represent transfection efficiency. For each experiment, N_o_ was determined from two timepoints on the curve, and the mean transfection efficiency reported.

## Results and discussion

The aim of this study was to compare the absolute and relative efficiencies of three electroporation-based transfection techniques. The standard techniques for direct electroporation of ring stage IE (transfections 1–3, Figure 1A, Protocol 1) and use of preloaded erythrocytes mixed with mature stage IE (transfections 4–6, Protocol 2) were compared with a novel “double-tap” technique that uses two “taps” of either 40 μg (transfections 7–9, Protocol 3) or 20 μg (transfections 10–12, Protocol 4) of plasmid. Using a plasmid bearing a luciferase reporter cassette, and an improved single-step lysis protocol to measure luciferase expression in small samples of IE, a quantitative monitoring of parasite growth can be readily employed to monitor the logarithmic growth of parasites that have successfully been transfected. Care was taken throughout the experiment to ensure as near as possible matched conditions for the transfection and subsequent outgrowth of cultures to facilitate a meaningful comparative analysis.

Examination of the timecourse of luciferase activity for these 12 experiments initially shows a background signal of some 30–50 relative light units (RLU), with an exponential increase in the luciferase signal from varying timepoints as the population of successfully transfected parasites grows, crosses the luciferase detection threshold and then continue to proliferate (Figure 1B). To allow the RLU measured to be correlated with parasite numbers, a standard curve was generated from a sequential dilution of the parasite clone Pfluc. Pfluc is a derivative of the AHEI clone where the pΔ1 plasmid has been integrated into the *cg*6 locus on chromosome 7. This standard curve (data not shown) plots log_10_ RLU against parasite number and shows a strong linear relationship (R^2^ 0.98) from a detection threshold of some 200 IE/40 μl sample to greater than 10^5^ parasites/40 μl sample. A non-linear exponential regression analysis of parasite numbers over time indicates the fold-increase in parasite number per cycle ranged from between 2.25-2.75, showing no correlation with the electroporation protocol employed. For this reason, the median value of a 2.5 fold increase in parasitaemia per cycle was adopted in all further calculations. Whilst resulting in all subsequent calculations being an approximation, the demonstration that growth rates across all the transfection experiments was similar, did provide important confirmation that efforts to ensure identical growth conditions post-transfection were effective.

Using all parameters measured (luciferase expression and microscopic confirmation of at least 1% parasitaemia), preloaded erythrocytes alone (transfections 4–6, Protocol 2) was the most efficient (mean efficiency of 9.59x10^-6^) protocol. All transfections recovered to at least a 1% parasitaemia by Day 17, which suggests that between 1,800-2,100 parasites were successfully transfected at the onset of the experiment. Whilst some parasites may have been transfected on subsequent reinvasion of a preloaded erythrocyte, the application of drug selection with 24 hours on Day 2 makes this unlikely. After preloaded erythrocytes, the “double-tap” and direct electroporation of ring IE alone show relative decreases in efficiencies of 5.6 and 184-fold, respectively. This comparison was made against transfections 1–3 (mean efficiency of 5.2x10^-8^, Protocol 1) and 10–12 (mean efficiency of 1.71x10^-6^, Protocol 4) which both use the same total amount (40 μg) of plasmid. Transfections 7–9 (“double-tap” of 40 μg plasmid) are almost twice as efficient (mean efficiency of 3.17x10^-6^, Protocol 3) as when a “double-tap” of 20 μg plasmid was used (mean efficiency of 1.71x10^-6^), which indicates under the parameters explored here a linear response in dose-dependent efficiency. Interestingly, the absolute efficiency of both “double-tap” protocols was much closer to that of the preloaded erythrocyte, which suggests that their success is largely attributed to the use of preloaded erythrocytes. It is likely that the measured decrease in efficiency is the result of a kill induced by the electrical current applied directly to the ring-stage parasites on Day 0.

Using the technique for routine quantitative monitoring of growth established here, other questions relating to the optimal conditions for electroporation-based transfections would appear to be simple to address in the future. For example, what concentration of preloaded plasmid DNA is optimal? Or, what is the optimal ratio of mature IE and preloaded erythrocyte? The most efficient transfection reported here of 1.08x10^-5^ needs to be significantly improved to overcome this “transfection bottleneck” should high throughput phenotypic studies of genetically-modified parasites become a routine tool. That said, recent improvements in transfection techniques, such as use of 96-well electroporation [14] and suspension cultures [15] go some way to address these limitations. In addition, there has been some success in increased transfection efficiency reported as a result of the sequential addition of preloaded erythrocytes over several days at the start of an experiment [7]. Whilst this was not explored here as the study was attempting a side-by-side comparison of techniques under the same starting conditions, evidence here from the “double-tap” transfections would suggest that this modification should be adopted more widely.

## Conclusion

This study provides a procedure to quantitatively assess both the relative and absolute transfection efficiencies of three electroporation protocols that clearly supports the use of preloaded erythrocytes. An exploration of the efficiency of a novel “double-tap” technique, combining previous standard protocols, shows that this technique is some six-fold less efficient than preloaded erythrocytes alone. The almost 200-fold increase in efficiency when preloaded erythrocytes are compared to direct electroporation of IE suggests that any transfection procedure that applies an electrical current directly to the IE parasite should be avoided and that this observation may have wider implications for the electroporation of any other intracellular organism.

## Competing interests

The authors declare that they have no competing interests.

## Authors’ contributions

PH and SH designed the study, analysed the data and prepared the manuscript. SH and KR carried out the experiments. All authors read and approved the final manuscript.
